# Demographic Factors and Trends Associated with Mortality After AIDS Diagnosis in Puerto Rico

**DOI:** 10.3390/idr18010013

**Published:** 2026-01-20

**Authors:** Grisel Burgos-Barreto, Daniel Reyes, Raymond L. Tremblay

**Affiliations:** 1 San Juan Bautista School of Medicine, Caguas 00726, Puerto Rico; 2 University of Arizona College of Medicine-Phoenix, Phoenix, AZ 85004, USA

**Keywords:** life years lost, HIV/AIDS, life expectancy, Puerto Rico

## Abstract

Background: Millions of people have died from AIDS-related illnesses since the start of the epidemic. The objective of this study is to determine the relationship between life years lost and demographic factors in the subset of individuals in Puerto Rico with advanced HIV disease, i.e., who received a diagnosis of AIDS, and to evaluate trends in poverty, age, and number of diagnoses and deaths over this timeframe. Methods: We identified 3624 individuals diagnosed with AIDS who received services under the Eligible Metropolitan Area (EMA) of San Juan, Puerto Rico, between 2000–2020, and correlated demographic factors with AIDS descriptive statistics using a retrospective cohort study design. We used socioeconomic characteristics to describe the population, estimated the life years lost (LYL) compared with the life expectancy of the general population of Puerto Rico at a given age as the null model, and evaluated the relationship of demographic variables with LYL, as well as trends in poverty and age/number of deaths/diagnoses over time. Results: More life years are lost with earlier AIDS onset, and there is also an association between LYL and the level of poverty, documented mode of transmission, and insurance status. LYL were higher among AIDS patients with lower income, with perinatal transmission, and among those without insurance in the age bracket of 40–49 years. No relationship between LYL and gender was detected. Moreover, over the years included in the timeframe of this study, certain trends emerged: we observed a greater proportion of AIDS to HIV diagnoses over time; HIV/AIDS diagnoses and deaths occurred on average at a higher age; the number of diagnoses per year initially rose over time and then declined; and the number of deaths per year as well as the poverty level in those diagnosed with HIV/AIDS increased over time. Conclusions: This study demonstrates the continued recent impact of the HIV epidemic specifically on those with advanced disease (AIDS), and further reaffirms the importance of treatment and prevention as well as demographic and social determinants of health, including age, poverty level, insurance status, and lifestyle, highlighting the disproportionate burden of HIV/AIDS among those with greater levels of poverty.

## 1. Introduction

Since the start of the epidemic in 1981, 42.3 million people died from illnesses related to AIDS, which is the late stage of infection of HIV [1]. AIDS is an advanced condition related to untreated HIV infection that is diagnosed based on specific AIDS-defining illnesses that are rare in the general population and tend to occur when circulating CD4+ lymphocytes are below <200/mm^3^ [2]. Advancements in diagnosis and in treatment of HIV, particularly antiretroviral therapy (ART), has led to prevention of progression to AIDS and a rise in life expectancy [3]. In 2024, 630,000 [490,000–820,000] people died from AIDS-related illnesses worldwide [1], and HIV/AIDS was the eleventh most common cause of disability-adjusted life-years globally in 2019, the second most common cause for individuals 25–49 years of age, and the ninth most common at 10–24 years of age [4].

Life years lost (LYL) is a metric for estimating disease impact that is calculated based on the age of onset or diagnosis of a condition [5] that overcomes the limitation of the traditional lifespan approach for diseases that can be acquired at different ages, and has been developed into an R package [6]. Average years of life lost (AYLL) describes the average number of LYL across all ages, to represent the number of additional years an individual would be expected to live if not for premature death from their disease versus the general population in their age group [7,8]. Whereas conventional mortality measures focus on the probability of death over a specified interval, LYL quantifies the average reduction in remaining life expectancy relative to the general population at a given age, thereby incorporating both whether and when death occurs. The method is more appropriate for conditions such as AIDS, where the age of onset can vary widely, and has previously been applied to other conditions [9,10,11], though not frequently in HIV/AIDS [12,13,14].

We previously calculated LYL in cohorts of patients with an HIV diagnosis without an AIDS diagnosis versus those with an AIDS diagnosis, regardless of prior HIV status [13], and also calculated LYL in those with an HIV diagnosis regardless of AIDS status [14] and analyzed the demographic variables in the latter population. For context, these prior analyses estimated LYL in a combined HIV/AIDS cohort using the same 2000–2020 San Juan EMA data and the same 1999–2001 Puerto Rico life table, in contrast to the present study which restricts the population for calculation of LYL to individuals with a documented AIDS diagnosis and a recorded date of death. Some prior studies estimating LYL in HIV/AIDS (not specifically for AIDS) have also been conducted in the mainland U.S. as well as outside the U.S. [12,15,16].

LYL related to AIDS diagnosis, the topic of this study, has been estimated on a still more infrequent basis. A 2014 study estimated burden of disease due to AIDS in the Brazilian Southern State of Santa Catarina at 257.5 years lost and 331.9 disability-adjusted life years (DALYs) per 100,000 total inhabitants, highest in males in the age groups of 30–44 and 45–59 [17]. A 2015 study in the same region estimated 593.1 DALYs/100,000 inhabitants (780.7 DALYs/100,000 men and 417.1 DALYs/100,000 women) among residents of the city of Tubarao, Santa Catarina State, Brazil, with men aged 30–44 years and women 60–69 years most affected [18]. Other studies looked at sociodemographic variables, such as a 2016 descriptive study [19] that found a difference between males and females who died from AIDS and AIDS-related causes (987 males and 340 females) among youth with secondary education in South Africa between 2009–2011, and a 2022 study analyzing the association of AIDS deaths in females between 2007–2017 in Porto Alegre in Rio Grande do Sul, Brazil, with race/skin and social vulnerability indicators. The latter study estimated 86.5 years lost/1000 females, with a higher proportion of deaths among females of White race/skin color (53.4%), but a higher rate of years of life lost among females of Black and “mixed” race/skin color living in regions with higher risk [20].

Various risk factors, such as poverty and transmission modality, have been correlated with greater LYL in HIV patients as a whole in our prior analysis [13]. In other studies, poverty has also previously been shown to be associated with decreased life expectancy among different age, gender, and racial/ethnic groups [21]. Risk factors, including different modes of transmission, have also been associated with differences in life expectancy [16,22]. Additionally, prior life expectancy estimates in Puerto Rico HIV patients have been generally lower than those from the mainland U.S., such as a 20-year-old in Puerto Rico having approximately 22 years to live [14], versus another study estimating a 21-year-old in the U.S. mainland having 54.9 years left [23], albeit with differing study parameters.

The estimation of life years lost allows the breakdown of specific contributors or causes of death at different ages of diagnosis, to evaluate the magnitude of each factor and help implement focused public health programs accordingly. Life years lost have not been frequently estimated for HIV/AIDS in general, and the association between LYL specifically after an AIDS diagnosis and sociodemographic variables has not yet been evaluated for the Puerto Rican population to our knowledge. We previously characterized and estimated LYL in the Puerto Rico HIV/AIDS population [13,14]. This included those HIV patients who received a diagnosis of AIDS, an advanced HIV condition that correlates with untreated disease. Whereas the earlier work examined LYL in a broader HIV cohort with and without AIDS, the present study focuses specifically on individuals with an AIDS diagnosis and investigates sociodemographic determinants and temporal trends in greater detail. The objective of the present study is to evaluate whether certain sociodemographic risk factors in patients with AIDS in Puerto Rico are associated with lower life expectancy as reflected in life years lost (LYL), as well as to detect trends such as level of poverty over time in this population that occurred during this period.

## 2. Methods

### 2.1. Study Design and Participants

This study employed a retrospective cohort design. Our study population included people diagnosed with AIDS who received services under the Eligible Metropolitan Area (EMA) of San Juan, Puerto Rico, during the period from 2000 to 2020, and who had a documented date of diagnosis of AIDS and a date of death. We described sociodemographic characteristics, including gender, poverty level (%), documented mode of transmission, type of health insurance, and age of AIDS diagnosis in the study population. Posterior to describing the population, we estimated LYL by estimating life expectancy in people diagnosed with AIDS and comparing with the general survivorship curves for Puerto Rico [24]. Subsequently, we calculated trends separately for various periods during the study for comparison.

We obtained the data for this study from electronic medical records of patients diagnosed with AIDS in the eligible metropolitan area (EMA) of San Juan, Puerto Rico, from 2000 to 2020. All information and data were kept strictly confidential, encoded with identification numbers, and stored only with those identification numbers. Access to coded or encrypted storage was physically limited to authorized personnel only via an access code. The San Juan Bautista School of Medicine IRB (EMSJBIRB-17-2022) approved the study (approved on 17 January 2023).

### 2.2. Data Analysis

The population of AIDS patients was described using frequencies and proportions by category. Out of 24,143 patient records associated with a diagnosis of HIV and/or AIDS, the initial survey size for this study was 3624 unique patients with a date of AIDS diagnosis. The sample size varies by analysis, and is noted in each case, since the information for some of the variables was not available. We only included patients who were diagnosed with AIDS and who had a date of death specified to be able to calculate the life expectancy, i.e., the average number of years between date of AIDS diagnosis and death. We then eliminated any patient data that appeared to be a duplicate (e.g., identical unique identifier and/or exactly identical birth/diagnosis/death dates) or had incomplete, unusable, or clearly incorrect information (e.g., missing or unusable data precluding valid LYL estimation such as date of death preceding diagnosis, or that is clearly incorrect due to physical impossibility such as date of death preceding date of birth).

The relative impact of AIDS on individuals’ means estimated life expectancy as a result of being diagnosed at a given age was estimated comparing with the null model for life expectancy of the survivorship curve of the 1999–2001 demographic survey for Puerto Rico [24]. Of note, evaluation of other survivorship curves (of other demographic surveys) showed only minimal differences and impact on the primary interpretation of the results. We selected the 1999–2001 Puerto Rico life table to maintain consistency with our prior HIV and HIV/AIDS LYL studies and because it provides stable estimates for the entire 2000–2020 period. To analyze of the impact of the number of “Life Years Lost: LYL” related to a disorder, we used a method proposed by Andersen (2017) [5] and Erlangsen et al. (2017) [25]. This model addresses the inherent challenge of estimating life span for age-specific mortality rates [5,6], estimating LYL from the time of initial diagnosis and comparing with the null model of the general population. The basic null hypothesis is that an individual who did not develop AIDS would, on average, have had the same life span as the general population. LYL therefore estimates the number of years of life, on average, to be lost by patients diagnosed at a given age. Similar methods have been previously described and used in other research [6,13,14].

Analysis was performed using R (version 4.3.0; R Core Team, 2023; R Foundation for Statistical Computing, Vienna, Austria) and Excel (version 16.72; Microsoft 2023; Microsoft Corporation, Redmond, WA, USA). We employed the “lilies” R package to estimate LYL, using the general population as our null distribution [26], and the “tidyverse” packages for wrangling and basic demographic and visualization tasks [27].

We performed analysis with a generalized linear model using a Gamma distribution to evaluate the association between life years lost and poverty level, and using a Gaussian distribution to evaluate the association between life years lost and insurance status, mode of transmission, and gender, with *p*-values less than 0.05 considered significant. We analyzed these factors in all patients who had available demographic information in these categories as well as an AIDS diagnosis date and a date of death. Regarding the poverty level, we used a Gamma distribution model to compare income in terms of the percentage of the national poverty level guidelines, which considers household income and family size [28], with years of life lost. Regarding insurance status, the status of being insured vs. uninsured and of having private vs. public insurance were each compared with average years of life lost (AYLL) in the given category. Transmission or risk factor categories were also compared with respect to AYLL both by age group and overall. With respect to individuals with multiple such categories, some individuals reported both MSM and IDU, so we created a combined MSM–IDU category to avoid double-counting. Other overlapping categories were not reported. Lastly, males versus females were compared, both by age group and overall.

Age-stratified summaries of LYL and AYLL such as by transmission category and insurance status are performed as descriptive, unadjusted comparisons intended to explore patterns across age groups rather than to provide fully adjusted effect estimates. These estimates are conditional on individuals with an AIDS diagnosis who died during follow up and had complete information available, rather than representing remaining life expectancy for all people diagnosed with AIDS in the EMA.

Additionally, to clarify terminology, ‘average years of life lost’ (AYLL) or average LYL refers to the average difference between expected lifespan in the general Puerto Rican population and lifespan after AIDS diagnosis, whereas ‘life expectancy after diagnosis’ denotes the mean years of life remaining after AIDS diagnosis among deceased individuals in this cohort.

## 3. Results

### 3.1. Population Characteristics

This study used a data set that included 3624 patients with a specific date of AIDS diagnosis, which was obtained from an initial data set of 24,143 patients with HIV/AIDS belonging to the eligible metropolitan area (EMA) of San Juan, Puerto Rico, from 2000 to 2020.

The average age of AIDS diagnosis was 42.8 years for men and 41.4 years for women. For transgender patients, it was 40.7 years. In this study, individuals had an average annual income of $6534. The majority of individuals (63.2%) lived at or below 50% of the poverty level. Most individuals (85%) had public health insurance (58% had Medicaid, and the remaining 27% had Medicare/other public insurance). Additionally, 7.86% had private insurance, 2.48% had “other” insurance, 4.5% were uninsured, and 0.06% had no specified insurance information. 47.1% of individuals in this study reported being presumably infected through heterosexual contact, 25.6% through the use of intravenous drugs, and 20.9% through male-to-male sexual contact. Only 1% reported both homosexual contact and intravenous drug use, while 1% reported transfusion, and 2% reported perinatal transmission. Table 1 presents the demographic information of the patients.

### 3.2. LYL Results

The mean life expectancy of the general population of Puerto Ricans of 80.25 years old (76.7 years old for males and 83.6 years old for females) is the mean life expectancy assumed by our model as the tau that we used for the maximum age required by the model for both males and females. We also tried varying the maximum tau between 80–99, with insignificant changes in interpretations of the main results.

### 3.3. Impact on AIDS Patients

The average expected LYL of all patients who were diagnosed with AIDS and already had a date of death (*n* = 505, after removing those with a date of death that preceded the date of AIDS diagnosis) is 28.56 overall (27.58–29.54), 54.75 years for the youngest patients (under 20) (35.87–73.63), 42.05 at age 30 (37.87–46.23), and 15.25 at age 60 (12.65–17.85). The average life expectancy across all ages (years of life remaining after the diagnosis) of an AIDS-diagnosed individual is between 5 and 8 years. The life expectancy of individuals with AIDS, irrespective of the age of onset, is many years less than that of the general population. An individual who is diagnosed with AIDS, at the age of 20, 30 and 50, has an expected mean lifespan of another ±9, 10 and 7 years, respectively. This is much less than the average life expectancy considering the whole population across all ages, which is ±40 years remaining to live for an age-matched cohort.

### 3.4. Poverty Level

**Figure 1:**
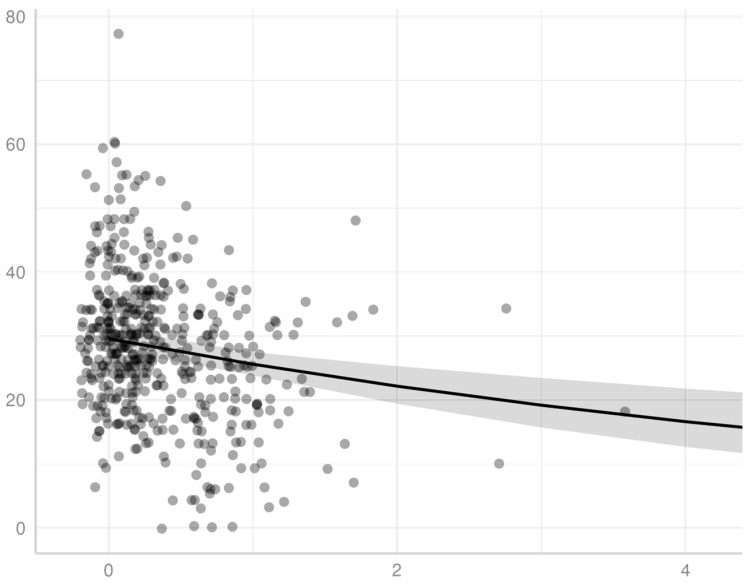
Mean LYL in years (y-axis) versus poverty level in terms of fraction of the federal poverty limit (x-axis) of individuals diagnosed with AIDS. The points represent individuals, and the line represents predicted values of LYL based on a gamma distribution model, including 95% confidence levels (shaded area). The threshold for 100% the federal poverty limit is equal to a value of 1 on the x-axis.

We demonstrated a significant inverse association for patients diagnosed with AIDS between income, measured by percentage of the poverty level, and LYL (*p* < 0.001), using a gamma distribution model. We observed this association model across all ages of AIDS diagnosis for patients in this study (Figure 1, as well as within specific age ranges of AIDS diagnosis, specifically for those diagnosed at the age ranges of 20–29, 30–39, 40–49, 50–59, and 60–69. An individual diagnosed with AIDS with income at <100% poverty level at the age of 35, 45, and 55 has an expected mean lifespan of approximately 44.4, 51.7, and 59.7, respectively. Of note, the latter set of ranges excludes the extremes of age, which had a smaller sample size and did not generate statistical significance, potentially related to lack of power. Extreme age ranges may also have less predictive accuracy with respect to both LYL as a measure of impact on survival (for example, in an elderly individual with a lower remaining life expectancy) as well as current household income as a measure of socioeconomic status (for example in a child or retired individual with no income).

### 3.5. Poverty Level over Time

**Figure 2:**
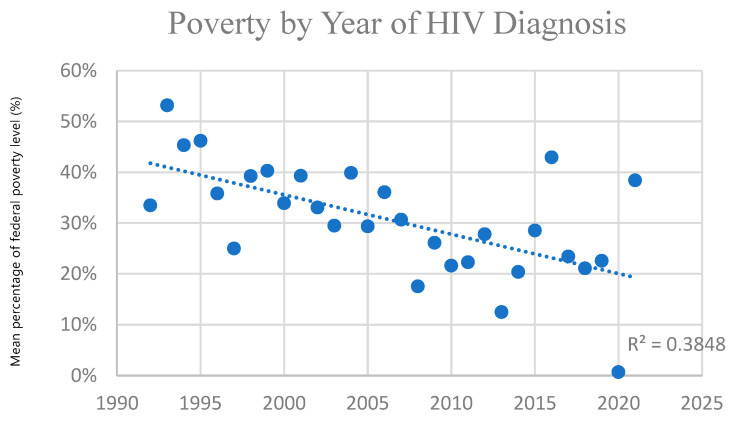
Poverty by year of HIV diagnosis: Linear regression of level of poverty, by percentage of the federal poverty limit (y-axis), of individuals diagnosed with HIV for whom LYL was calculated (total *N* = 1036), as a function of calendar year of diagnosis (x-axis). Each point is based on all individuals diagnosed in that specific calendar year for whom LYL could be estimated.

**Figure 3:**
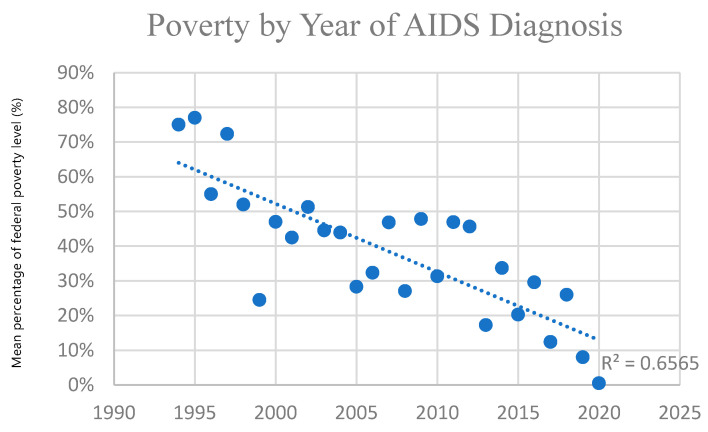
Poverty by year of AIDS diagnosis: Linear regression of level of poverty, by percentage of the federal poverty limit (y-axis), of individuals diagnosed with AIDS for whom LYL was calculated (total *N* = 505), as a function of calendar year of diagnosis (x-axis). Each point is based on all individuals diagnosed in that specific calendar year for whom LYL could be estimated.

Poverty level (based on income when the patient was alive) increased over time in those diagnosed with AIDS in this cohort (in those for whom LYL was estimated), indicating progressively higher poverty among AIDS diagnoses over the study period. This trend was greater in those with AIDS than those with HIV generally, who also demonstrated this trend. Figure 2 and Figure 3 below demonstrate this significant trend using one-year windows. Those diagnosed with HIV before 2010 had an average income at 40% of the poverty limit, versus 20% after 2010. (*p* = 0.0003) Those diagnosed with AIDS before 2010 had an average income at 40% of the poverty limit, versus 30% for those diagnosed after 2010. (*p* = 0.03) The trend for those diagnosed <1990, 1990–2000, 2000–2010, and 2010–2020 was 40%, 40%, 30%, and 20%, respectively, for HIV, and 170%, 50%, 40%, and 30% for AIDS. This indicates greater levels of poverty for those diagnosed with HIV and AIDS over time, which is more pronounced in those with AIDS. Thus, economic disparity has increased with AIDS in particular becoming more and more a disease affecting the poor (Figure 2 and Figure 3). The overall poverty trends were similar when stratified by gender, although cell sizes in some year–gender strata were small.

### 3.6. Mode of Transmission

**Figure 4:**
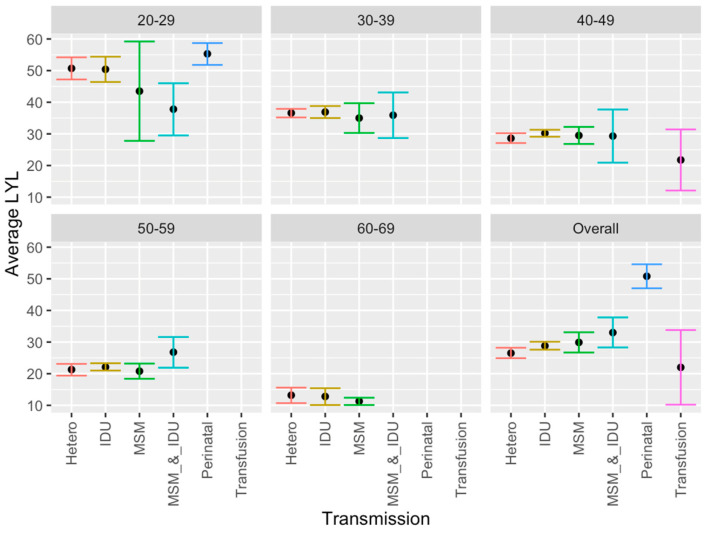
Average LYL by mode of transmission type of individuals diagnosed with AIDS, including 95% confidence levels and the age of first diagnosis.

Significantly greater LYL were found across all ages of patients diagnosed with AIDS with perinatal transmission compared to other modes of transmission, and greater LYL were found with IDU and MSM combined compared with heterosexual contact, in descriptive age-stratified analyses. Other differences were observed within specific age groups. Specifically, in the age group 20–29, IDU alone had greater LYL than IDU and MSM combined (Figure 4). However, as a general pattern LYL was generally similar among transmission methods within a given group age category.

### 3.7. Medical Insurance

There was no significant difference found between insurance categories for LYL when calculated across all ages for AIDS patients in this study. Although greater LYL were found in those with no insurance (34.6) versus those with insurance (29.5), the difference was not statistically significant (*p* = 0.13).

Among individuals whose age at diagnosis fell within the range of 40–49, however, we did demonstrate significantly greater LYL were found in those with no insurance versus with insurance (34.3 versus 29.2, respectively, *p* = 0.04). All age groups exhibited this general trend, but it was only significant in the 40–49 age range, which also included the greatest number of patients (*n* = 179 with insurance, 7 without). Among the age group of 40–49 years, those with no insurance also had significantly greater LYL than those with public insurance or Medicaid specifically. No significant difference in LYL was observed between public versus private insurance in any age group, nor overall. We also found no significant difference between specific types of public insurance (e.g., Medicaid versus Medicare or other public insurance).

### 3.8. Gender

**Figure 5:**
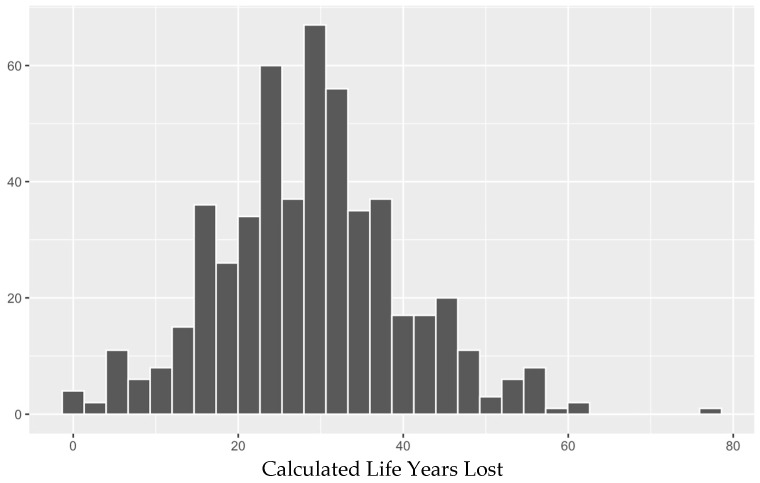
Frequency distribution of LYL for all deceased individuals diagnosed with AIDS, irrespective of the age of diagnosis.

We found no association with gender, with approximately equal LYL found overall, and within all age groups, between males and females with AIDS. This was also previously found to be the case with males and females with HIV generally [14]. Overall, patients lost a total of 28.6 years (28.4 ± 1.2 for males, and 28.9 ± 1.8 for females). Transgender patients and those who refused to answer the question regarding gender tended to have greater AYLL, but these were few in number (*n* = 1 and 1, respectively). In general, as we previously found for HIV/AIDS patients [13,14], number of life years lost decreased with increased age at AIDS diagnosis. Of note, our use of a Gaussian distribution for comparison of LYL is justified, as LYL values followed a normal distribution (Shapiro-Wilks test of normality, *p* > 0.05; see Figure 5 below).

### 3.9. Trends over Time in AIDS Patients

**Figure 6:**
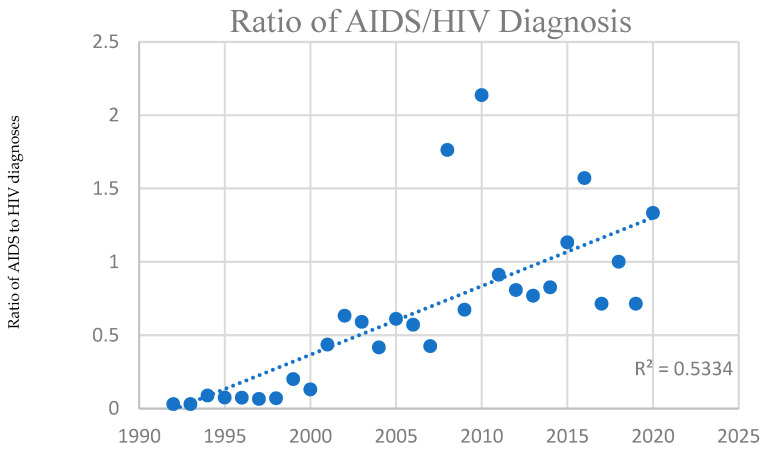
Ratio of AIDS/HIV diagnosis: Linear regression of ratio of AIDS to HIV diagnoses (y-axis) for patients included in the LYL calculations as a function of year of diagnosis (x-axis), with each point representing the ratio of number of diagnoses of AIDS to HIV in patients for whom LYL was calculated for that particular year.

Over the years of this study, AIDS diagnoses were made on average at a greater age in later years compared with earlier years of this data set, and was also at a greater age than for HIV (42.6 ± 1.1 prior to 2010 versus 49.6 after 2010 ± 1.6 for AIDS diagnoses, versus 38.3 ± 0.7 prior to 2010 versus 48.3 ± 1.8 after 2010 for HIV diagnosis). Additionally, deaths occurred on average at a higher age during later years (e.g., recorded deaths in this population of HIV patients tended to occur on average between 42–50 years of age through 2012, then between 51–60 from 2013–2022). Diagnoses themselves initially increased and then decreased while deaths increased. The proportion of AIDS to HIV diagnoses increased over time, as shown in Figure 6 below using one-year time windows. As noted above, poverty level in those diagnosed with AIDS trended to increased poverty over time, suggesting an increasingly disproportionate effect on those without means.

## 4. Discussion

Various demographic factors are associated with LYL among AIDS patients. Age of diagnosis carries an association with LYL, as previously demonstrated among HIV patients [14], and as expected, given there are fewer years to lose with age. A newborn diagnosed with AIDS is likely to lose approximately 40 years of life, while a 50-year-old is expected to lose closer to 20 years of life. This trend is consistent with other studies estimating LYL in the United States [16,29]. AIDS diagnoses are associated with greater LYL than HIV diagnoses, despite generally occurring at a later age. On average across all ages, those diagnosed with AIDS had 5–8 years left to live, versus those diagnosed with HIV, who had 12–14 years left. Compared to the average 31.67 life years lost following HIV diagnosis at age 30 [13], those diagnosed with AIDS at age 30 lost 42.05. Greater LYL for AIDS vs. HIV patients overall were also exhibited within the most impoverished segments of the population, where those with AIDS below 100% of the poverty level at the ages of 35 and 45 had an expected mean lifespan of approximately 44.4 and 51.7 respectively, versus the HIV population overall below 100% of the poverty level with an expected mean lifespan of 51.2 ± 2.8 and 56.2 ± 2.5 years, respectively [14]. Diagnosis at later stages of disease may allow for less control of the disease and greater LYL as a result. This finding is consistent with prior findings looking at those with HIV who never developed AIDS versus all those who developed AIDS [13], and also consistent with previous results in 2016 [16] indicating that those with late-stage disease at diagnosis (stage 3, i.e., AIDS) had a life expectancy that was, on average, 6.6 years lower than that for persons with HIV who never received a diagnosis of AIDS (stage 3 HIV). This indicates poorer outcomes with later diagnosis and more advanced/uncontrolled disease. 5.9 million people did not know they were living with HIV in 2021, per UNAIDS data [1], signifying the prevalence of those who are not aware that they were infected initially and are at risk of progressing to later stages of the disease before diagnosis and treatment.

Furthermore, we found certain risk factors to be associated with increased mortality, as well as trends associated with this. We noted not only a significant association with poverty level, where poverty is associated with greater LYL, but that AIDS diagnoses in those for whom we calculated LYL have tended to occur in poorer populations over time. This signifies that this is becoming more and more a disease of the poor over time, particularly as treatments such as ART have been available to reduce, if not eliminate, the increased mortality in this population. This is further highlighted by the more pronounced trend associated with AIDS diagnoses versus HIV, suggesting that those who are diagnosed late or are found to have already progressed to AIDS when they are first discovered to be HIV positive tend to be those with less access to healthcare resources and in more underprivileged segments of society, even when as a whole our population tended to have high levels of poverty. There are limitations in this analysis, however, where these observed poverty trends may also be influenced by or reflect other factors such as cohort effects, changes in HIV testing and linkage to care, survivorship or selection bias in who remains in EMA services, and potential changes in reporting or eligibility criteria over time.

Certain modes of transmission were also associated with greater LYL than others. Although potentially expected given the association with age, significantly greater AYLL were found following perinatal transmission compared with other modes of transmission. Greater LYL were also found in the IDU and MSM combined group versus heterosexual contact for patients overall, which was significant across patients of all ages combined, though not significant within any age category, likely due to lower power within the individual age categories. The greater years lost with IDU and MSM likely relate to rates of transmissibility of the virus and other infections with intravenous injection and male-to-male sexual contact, and/or association with risky behaviors and other risk factors such as lower socioeconomic status in the injection drug group. It is also hard to know with certainty how the disease was transmitted in a given individual when multiple modes of transmission are possible. In addition, we are assuming accurate reporting of sexual behavior represented in the data set, despite the stigma still associated with various sexual behaviors, which may reduce this accuracy [30]. Moreover, acceptable social behavior and associated stigma may also vary between age groups as well as across timeframes.

We found greater LYL in individuals without insurance versus those with insurance across all age groups, although this difference was only significant in the age range of 40–49 years. This age range had the highest number of patients and thus greater power. Other factors could also play a role, such as the fact that this age group may have been more likely to have insurance tied to employment, versus children or young adults, whose insurance may be through parents, school, or another entity, or older patients who are eligible for Medicare. No significant difference in LYL was detected between public versus private insurance in any age groups, which may be due to no actual difference, or due to inadequate reporting or lack of power leading to a type II error. This is interesting since U.S. Medicaid insurance has previously been shown not to be equivalent in services to private insurance [31]. Of note, we have also observed the above trends concerning insurance in our prior analysis involving HIV patients as a whole, with or without AIDS [14].

Because insurance status is intertwined with age, employment, Medicaid/Medicare eligibility, and potentially calendar time, the observed differences in LYL by insurance category may partly reflect these underlying structural and policy factors rather than insurance alone, and residual confounding by such determinants is likely. The relationship with LYL is likely complex/multifactorial, and/or these could serve as confounding factors, as is often the case given the inherently interconnected nature of sociodemographic variables. Accordingly, we interpret insurance-related LYL differences cautiously as indicators of broader structural disadvantage, though treatment and access to care generally appear to play a critical role given the known effectiveness of ART on improving mortality [23,24].

Many of our results are generally consistent with prior studies. Poverty has been associated with lower life expectancy in different age, gender, and racial/ethnic groups [17], and socioeconomic, racial, and geographic disparities in HIV/AIDS have persisted with higher HIV/AIDS mortality in more socioeconomically deprived groups [32]. Transmission/risk factor categories have also been analyzed, with the MSM category being found to have a longer life expectancy than other groups in some studies [16,18], though we did not detect this trend. We also did not detect gender differences in this study on AIDS patients nor in prior analyses on HIV patients as a whole [13,14]. However, other studies did find gender differences with regard to AIDS life expectancy specifically [13,19,20], as well as HIV generally in one study [23] where it was also found in the mainland US that adults with HIV have similar life expectancy to the general population, which also contrasts with our results in Puerto Rico. This difference does not appear to be related to sample size but instead appears to be specific to our demographic in Puerto Rico, as our study was adequately powered and demonstrated nearly identical LYL between genders overall across all age groups as well as within each age group. Nonetheless, gender-specific differences reported in larger U.S. cohorts warrant caution in generalizing our null finding. The discrepancy may reflect contextual differences in the Puerto Rican HIV/AIDS sociodemographic and care environment as well as residual confounding that we could not fully address.

Though our dataset did not include therapeutic information, the fact that life expectancy approximates baseline in adequately treated HIV suggests that inadequate ART treatment access, quality, and adherence is the ultimate downstream reason for the mortality differential, with social determinants of health such as those we examine likely at the root of the disparity.

Our study is limited in that the sample sizes are unequal among ages, as the number of newborns, youth, and those aged 60 or above diagnosed with AIDS was small. Additionally, the number of recorded transgender individuals in the cohort was very small, with only one transgender patient contributing complete data to the LYL analysis. This could be related to underreporting or incomplete information due to factors such as gender being recorded based on birth sex or being reported by patients as binary based on stigma or for privacy reasons. We consequently could not generate meaningful estimates of life expectancy or LYL for transgender people with AIDS, underscoring the need for larger/focused studies in this population.

Additionally, the deaths reported during the study period by the San Juan Eligible Metropolitan Area (EMA) were not matched with the Puerto Rico Demographic Registry. Moreover, the data for the San Juan Eligible Metropolitan Area (EMA) does not include people who have been tested anonymously or people infected with HIV/AIDS who were not tested. The potential for incomplete death ascertainment presents a limitation because deaths recorded in the EMA database were not systematically matched to the Puerto Rico Demographic Registry. If deaths among people with AIDS were missed or occurred outside the EMA system, survival time after AIDS diagnosis could be overestimated and life years lost underestimated, particularly for patients who moved, were lost to follow-up, or had limited access to care. Conversely, any differential under-reporting by age, socioeconomic status, or transmission category could bias comparisons between groups and partially obscure true mortality disparities.

An additional limitation is that LYL is estimated only among deceased individuals and had complete information, rather than among all 3624 people diagnosed with AIDS. As a result, our estimates are conditional on death and may overestimate years of life lost compared with the entire AIDS-diagnosed population, particularly if survivors differ systematically from decedents in age, comorbidities, or access to care. Accordingly, the LYL values presented here should be interpreted primarily as comparative indicators across sociodemographic groups, not as unbiased population-level projections of remaining life expectancy after AIDS diagnosis.

Currently, another limitation is in the ability of our estimated values for LYL to be compared with prior studies, given the lack of prior studies using the same time frames, methods, and populations. For example, few studies include Puerto Rico (e.g., a 2016 U.S. study [16] and a 2010 U.S. study [29]) and therefore will likely produce different results due to the different population and access to care in the mainland United States versus Puerto Rico, in addition to different time frames and methods. Additionally, methods may vary (e.g., using a life table from a different year). The use of more recent data may also yield different results, with mortality potentially improved due to developments in treatment [16] that can help improve life expectancy, though not without co-morbidities [23]. However, HIV mortality in the United States mainland [23] is substantially different from that in Puerto Rico, likely reflective of socioeconomic, racial, and geographic disparities [32]. Nonetheless, caution is needed in comparing different studies with different methods that may estimate different quantities, and researchers should consider the purpose of the research and the type of available data when deciding among methods to estimate LYL [7].

Data limitations also exist, such as restrictions in categorization or limitations in knowledge to assign categories. For example, it may be challenging to determine with certainty the mode of transmission, particularly when more than one potential mode or risk factor exists. We combined MSM and IDU but did not include other combinations due to lack of availability of such data. The combined MSM and IDU group also reduced the number of patients in the individual MSM and IDU groups because we did not include individuals in more than one overlapping group to avoid double counting, although the number in this combined group was relatively small. In addition, while the effect is likely minimal, the treatment of overlapping transmission categories (e.g., the small MSM–IDU combined group and the exclusion of other overlaps) may lead to some misclassification and biased comparisons between risk groups, because true patterns of multiple risk behaviors cannot be perfectly represented with the available data.

A further limitation is that our age-stratified comparisons (for example, by transmission category and insurance status within age groups) were not adjusted for other or interacting sociodemographic variables. These age-specific results should therefore be interpreted as descriptive patterns that may reflect residual confounding by factors such as poverty level, calendar period, and other unmeasured covariates, rather than as fully adjusted causal estimates.

Additionally, LYL was estimated using a single 1999–2001 Puerto Rico survivorship curve and a fixed maximum age for all diagnoses from 2000–2020, which may not fully reflect temporal improvements or sex-specific differences in background mortality; this could slightly misstate absolute LYL values, although as noted above, exploratory variation of tau did not materially change the relative patterns across subgroups. This choice may slightly overestimate absolute LYL in later calendar years due to temporal improvements in background mortality, but our main inferences focus on relative differences across sociodemographic groups.

Finally, the dataset did not include systematically coded information on non-HIV comorbidities such as cardiovascular disease, malignancies, or substance use disorders. We were therefore unable to quantify the contribution of these conditions to LYL or to adjust for them in our models. Because comorbidities are likely to cluster with sociodemographic factors such as poverty and insurance status, residual confounding by unmeasured comorbid disease burden may influence some of the observed associations.

In conclusion, we estimated average years of life lost to evaluate the impact of an AIDS diagnosis in Puerto Rico and evaluated associated risk factors. The number of life years lost (LYL) in AIDS patients is related to age of onset as well as to insurance/no insurance status, poverty level, and transmission mode, but not male versus female gender, and AIDS is becoming more of a disease of the poor with time. Additionally, those with AIDS have fewer years of life to live than those with HIV, despite AIDS being diagnosed later than HIV and an inverse association with age and LYL. This study has important implications regarding sociodemographic disparities in HIV/AIDS mortality.

## Figures and Tables

**Table 1:** Demographic characteristics of AIDS patients who received services under the Eligible Metropolitan Area (EMA) of San Juan, Puerto Rico, from 2000 to 2020, the primary population from which our study population was drawn.

Characteristic	N	Percent
Total	3624	100.00
Vital Status Alive Dead Unknown	3624 2615 604 405	100.00 72.16 16.67 11.18
Gender Male Female Transgender No answer Unknown	3624 2386 1230 7 1 0	100.00 65.84 33.94 0.19 0.03 0.00
Age (years) at diagnosis 0–9 10–19 20–29 30–49 40–49 50–59 ≥60	3624 22 36 382 977 1282 719 206	100.00 0.61 0.99 10.54 26.96 35.38 19.84 5.68
% Poverty Level ≤50 >50% ≤ 100% >100 “N/A”	3624 2291 713 432 188	100.00 63.22 19.67 11.92 5.19
Type of Medical Insurance Public Medicaid Medicare Other public Private Other Uninsured Unknown	3624 3084 2118 311 655 285 163 90 2	100.00 85.10 58.44 8.58 18.07 7.86 2.48 4.50 0.06
Mode of transmission Heterosexual Intravenous Drug Use (IDU) Men Sex Men (MSM) Perinatal IDU & MSM Blood transfusion Other Hemophilia Not specified	3624 1705 926 756 74 43 36 23 3 58	100.00 47.05 25.55 20.86 2.04 1.19 0.99 0.63 0.08 1.60

## Data Availability

Data presented in this study may be made available on request from the corresponding author with the permission of the AIDS Task Force.

## Abbreviations

The following abbreviations are used in this manuscript:

AIDS	Acquired Immunodeficiency Syndrome
ART	Antiretroviral Therapy
AYLL	Average Years of Life Lost
EMA	Eligible Metropolitan Area
HIV	Human Immunodeficiency Virus
MSM	Men who have Sex with Men
IDU	Intravenous Drug Use
LYL	Life Years Lost
PR	Puerto Rico
